# Three Novel de Novo *SOX4* Variants Expanding the Phenotypic Spectrum: Case Series and Literature Review

**DOI:** 10.3390/genes17091011

**Published:** 2026-08-26

**Authors:** Haixia Miao, Ting Zhang, Kexin Fang, Shuai Chen, Xiaocha Xu, Yi Zhang, Xinwen Huang

**Affiliations:** Department of Genetics and Metabolism, Children’s Hospital, Zhejiang University School of Medicine, National Clinical Research Center for Children and Adolescents’ Health and Disease, Hangzhou 310051, China

**Keywords:** nystagmus, hypospadias, whole-exome sequencing

## Abstract

Background: Variants in *SOX4* cause intellectual developmental disorder with speech delay and dysmorphic facies (IDDSDF), an autosomal dominant disorder characterized by global developmental delay, mild-to-severe intellectual disability, speech delay, distinctive facial features, digital anomalies, congenital heart defects (unique), and behavioral abnormalities. To date, only a limited number of patients have been described and the phenotypic spectrum of this disorder has yet to be fully delineated. Methods: Clinical data from three patients were collected, including clinical features, growth and developmental profiles, neuropsychological assessments, and urogenital evaluations. Whole-exome sequencing (WES) was performed to screen for candidate variants, and variant pathogenicity was interpreted following the American College of Medical Genetics and Genomics (ACMG) guidelines. Results: All three patients carried previously unreported de novo truncating variants in *SOX4*: c.583C>T (p.Gln195*), c.1347del (p.Cys450Alafs*5), and c.153G>A (p.Trp51*). Regarding the clinical phenotypes, Patient 3 (P3) was similar to previously reported cases, whereas Patient 1 (P1) and Patient 2 (P2) each exhibited distinctive features on the basis of overlapping with previous reports: P1 presented with severe hypospadias accompanied by cryptorchidism; to our knowledge, no case with this predominant clinical feature has been reported previously. P2 exhibited nystagmus, a novel phenotype not yet reported in the literature. Conclusions: We report three previously unreported de novo truncating variants in *SOX4*, expand the phenotypic spectrum of *SOX4*-related disorders to include nystagmus for the first time, and document one patient presenting with severe hypospadias and cryptorchidism. Our findings further expand the mutational and clinical phenotypic spectra of *SOX4*, underscore the marked clinical heterogeneity of this disorder, and provide new evidence to facilitate clinical recognition and genetic counseling.

## 1. Introduction

The *SOX* family consists of 20 members, which are classified into eight subgroups (A to H) according to sequence conservation. Among them, *SOX4*, together with *SOX11* and *SOX12*, belongs to the SOXC subgroup and plays critical roles in regulating progenitor cell development and determining the genetic programs of differentiated tissues [1]. SOX proteins primarily regulate gene transcription through interactions with partner proteins, either by heterodimerizing with other SOX members or distinct transcription factors, or by acting as homodimers [2]. Pathogenic variants in approximately half of the 20 human *SOX* genes have been associated with rare congenital disorders, collectively termed SOXopathies [3]. For instance, pathogenic variants in *SOX11*, *SOX9*, *SOX5*, and *SOX10* are responsible for Coffin–Siris syndrome 9 (CSS9, MIM:615866), campomelic dysplasia (MIM:114290), Lamb–Shaffer syndrome (MIM:616803), and Waardenburg–Hirschsprung disease (MIM: 613266), respectively.

As core members of the SOXC subgroup, *SOX4*, *SOX11* and *SOX12* play critical roles in multiple developmental pathways and exhibit partial functional redundancy. SOXC-related disorders share considerable phenotypic overlap with Coffin–Siris syndrome (CSS, MIM: 135900), which is characterized by hypotonia, global developmental delay, intellectual disability, coarse facial features, hypoplasia or aplasia of the distal phalanges of the fifth digits, and hypertrichosis [4,5,6]. Most CSS-causative genes encode subunits of the BAF (BRG1/BRM-associated factor) chromatin remodeling complex, and thus CSS is also categorized as a BAFopathy. Notably, *SOX4* and *SOX11* are transcriptional targets of the BAF complex, suggesting a potential molecular link between SOX4-related disorders and CSS [7].

In 2019, Zawerton et al. first reported four patients with de novo heterozygous missense variants in *SOX4*, all located within the highly conserved HMG domain of the SOX family. In vitro functional assays demonstrated that these variants abolished DNA binding and transcriptional activation by the SOX4 protein. All four patients presented with developmental delay, intellectual disability, and mild facial and digital anomalies. This study established for the first time that missense variants in the *SOX4* HMG domain cause a distinctive human neurodevelopmental disorder [8]. On the basis of the phenotypic overlap with CSS and the molecular evidence that *SOX4* is a downstream target of BAF complex, SOX4-related disorders were designated as Coffin–Siris syndrome 10 (CSS10, OMIM: 618506). However, Angelozzi et al. described 17 patients with *SOX4* variants in 2022 and challenged the validity of the CSS10 nomenclature. Although the clinical manifestations of these patients partially overlapped with CSS, they lacked the hallmark CSS features, including fifth-nail hypoplasia/aplasia, hypertrichosis, and corpus callosum agenesis [7]. Currently, the OMIM database has renamed this condition “Intellectual developmental disorder with speech delay and dysmorphic facies” (IDDSDF), with CSS10 retained as an alternative synonym.

As of December 2025, only approximately 40 patients with coding-sequence variants in *SOX4* have been reported, originating from 29 unrelated families and involving a total of 29 distinct variants. Both the case number and variant spectrum of this disease remain limited. Affected patients commonly present with core phenotypes, including neurodevelopmental delay, intellectual disability, and mild facial dysmorphism, whereas other manifestations exhibit considerable inter-individual variability [6,7,8,9,10,11,12]. In this study, whole-exome sequencing (WES) was performed to identify three novel heterozygous variants in *SOX4* from three unrelated probands, confirming the genetic basis of their conditions. Clinical phenotyping revealed two unreported key manifestations: nystagmus was identified as a novel phenotypic feature of SOX4-related disorders for the first time, and one proband exhibited severe hypospadias accompanied by cryptorchidism as the predominant clinical presentation. These findings further expand the mutational and phenotypic spectra of SOX4-associated disorders, providing reliable evidence for clinical management and genetic counseling of this rare disease.

## 2. Materials and Methods

### 2.1. Patients

Three unrelated patients with pathogenic *SOX4* variants detected by WES were retrospectively enrolled in this study. Patients were recruited from the Children’s Hospital of Zhejiang University School of Medicine during the period from January 2022 to December 2025. For each proband, comprehensive clinical data were systematically collected, including clinical features, developmental status, neuropsychological assessment results, and urogenital system evaluations. Before embarking on genetic analyses, we obtained signed informed consent from the participants’ guardians. This study received ethical approval from the Institutional Review Board of the Children’s Hospital, Zhejiang University School of Medicine, approval code 2020-IRBAL-035.

### 2.2. DNA Isolation

To enable genetic investigation, 2 mL of peripheral venous blood was collected from each patient and their parents. Purification of genomic DNA was performed using the MagBio Blood Spots Genomic DNA Purification Kit (Bioer Tech., Hangzhou, China), following the manufacturer’s standard operating procedure. Both the quantity and purity of the resulting DNA were evaluated using the Qubit dsDNA HS Assay Kit (Yeasen, Shanghai, China). Acceptance into downstream analytical workflows was contingent upon meeting these quality criteria: a total DNA amount of at least 200 ng, a concentration above 20 ng/μL, and an A260/280 absorbance ratio between 1.7 and 2.0.

### 2.3. Whole-Exome Sequencing 

Exome enrichment was performed for all index patients and their parents using the KAPA HyperExome V2 Probes Kit (Roche Molecular Systems, Inc., Pleasanton, CA, USA), which captures all protein-coding exons along with surrounding intronic sequences. The prepared libraries were then sequenced using 150-bp paired-end reads on the MGI DNBSEQ-T7 platform (MGI Tech., Beijing, China), producing an average coverage of greater than 120×, with over 98.5% of targeted bases reaching a depth of at least 20×. Following quality control, reads that met the criteria were aligned to the GRCh37/hg19 reference assembly using BWA (version 2.2.1). Single-nucleotide alterations and short insertion–deletion events were called using GATK version 4.0. The identified variants were subsequently characterized and categorized through VEP (release 113) and ANNOVAR (dated 20250302). Population-level allele frequencies were extracted by querying gnomAD (version 2.1.1; https://gnomad.broadinstitute.org; accessed on 1 August 2025) and dbSNP (http://www.ncbi.nlm.nih.gov/snp/; accessed on 19 June 2025). Conservation profiles, amino acid residue changes, and structural consequences were assessed using OMIM (http://www.ncbi.nlm.nih.gov/omim; accessed on 20 November 2025), ClinVar (http://www.ncbi.nlm.nih.gov/clinvar/; accessed on 1 August 2025), and HGMD (http://www.hgmd.org/; accessed on 26 September 2025).

The variant prioritization process began with the removal of frequent alleles, applying a threshold of MAF > 2% or AN > 2000 in gnomAD (v2.1.1; https://gnomad.broadinstitute.org; accessed on 1 August 2025), together with any variants recorded in our internal repository. Loci classified as benign in ClinVar (https://www.ncbi.nlm.nih.gov/clinvar; accessed on 1 August 2025) were subsequently eliminated, except those carrying prior designations of pathogenic, likely pathogenic, uncertain significance, or conflicting interpretation. Additional culling removed variants not listed in HGMD (http://www.hgmd.org/; accessed on 26 September 2025) and those yielding a SpliceAI delta score below 0.5 (https://spliceailookup.broadinstitute.org; accessed on 19 July 2025). The remaining variants were then prioritized through a multi-dimensional framework incorporating: (i) phenotypic concordance; (ii) inheritance patterns, with preference given to de novo (autosomal dominant), homozygous (autosomal recessive/X-linked), compound heterozygous (autosomal recessive), autosomal recessive, and X-linked recessive models; (iii) pathogenicity support, as integrated from HGMD, ClinVar, SIFT, REVEL, and SpliceAI; and (iv) sequencing quality parameters, encompassing mutant allele fraction (VAF) and depth of coverage. Definitive selection of putative variants hinged on an integrated appraisal of the above standards.

### 2.4. Sanger Sequencing

Upon completion of variant screening and prioritization, Sanger sequencing was employed to validate the selected candidate variants. Polymerase chain reactions (PCRs) were set up in 25 µL volumes, each containing 50 ng of template DNA, 1 µL forward primer and 1 µL reverse primer (10 pmol each), 12.5 µL of 2×Phanta Max Master Mix (Vazyme, Nanjing, China), and nuclease-free water. The cycling conditions comprised an initial denaturation step at 95 °C for 2 min; then 36 cycles of 95 °C for 30 s (denaturation), 58 °C for 30 s (annealing), and 72 °C for 1 min (extension), followed by a final elongation at 72 °C for 2 min. Amplified fragments were first examined via 1.5% agarose gel electrophoresis and subsequently subjected to capillary electrophoresis on an ABI 3730xl DNA Analyzer (Applied Biosystems, Foster City, CA, USA). Raw sequencing traces were analyzed using DNASTAR MegAlign software (version 3.3.8; DNASTAR, Inc., Madison, WI, USA).

### 2.5. Literature Search Strategy

A systematic literature search was conducted in the PubMed database (https://pubmed.ncbi.nlm.nih.gov (accessed on 26 July 2026)) from inception to 31 December 2025. The search strategy employed the following terms: “*SOX4*”, “*SOX4* mutation”, “Coffin–Siris syndrome 10”, and “intellectual developmental disorder with speech delay and dysmorphic facies (IDDSDF)”. We included case reports and case series that documented patients carrying protein-coding variants in *SOX4* with comprehensive clinical phenotypic descriptions. Studies focusing on tumor somatic variants and copy-number variations (CNVs) were excluded to accurately characterize the clinical and mutational spectra of germline *SOX4* variants.

## 3. Results

### 3.1. Clinical Features

The three patients were recruited from three unrelated families, and no parental consanguinity was reported in any family. All patients were born at term; one had low birthweight, while the other two infants had birth weights appropriate for their gestational age. The mean age at presentation was 9 months and 17 days, with height and weight ranging between −1 SD and −3 SD (Table 1). 

Patient 1 (P1) presented with severe hypospadias, cryptorchidism, and delayed language development, with no dysmorphic facial features. The external masculinization score (EMS) was 8, and the Prader stage was 3.

Patient 2 (P2) had distinctive facial features, including frontal bossing, flat nasal bridge, left esotropia, and a left single transverse palmar crease. Neurologically, the patient presented with nystagmus, hypotonia, unsteady gait with frequent falls, and delayed language development. The patient failed the left-ear hearing screen. Echocardiography revealed mild tricuspid regurgitation. Brain MRI demonstrated mildly widened bilateral frontotemporal extra-axial spaces and mild enlargement of the left lateral ventricle. Fundus screening showed no abnormalities.

Patient 3 (P3) presented with delayed language development. Echocardiography revealed a ventricular septal defect (VSD) with mild mitral and tricuspid regurgitation. At 3 months of age (May 2025), she was assessed using the Peabody Developmental Motor Scales, Second Edition (PDMS-2). Standard subtest scores for gross motor function were 3 for reflexes, 1 for stationary, 2 for locomotion, and 2 for object manipulation; fine motor subtest scores were 3 for grasping and 2 for visual–motor integration. All PDMS-2 subtest scores were below the 5th percentile of age-matched normative values. At 1 year of age (February 2026), evaluation using the Gesell Developmental Schedule yielded the following developmental quotient (DQ) scores: 79 for adaptive behavior, 68 for gross motor skills, 78 for fine motor skills, 76 for language ability, and 70 for personal–social behavior, indicating global developmental delay.

### 3.2. Sox4 Variants

**Figure 1:**
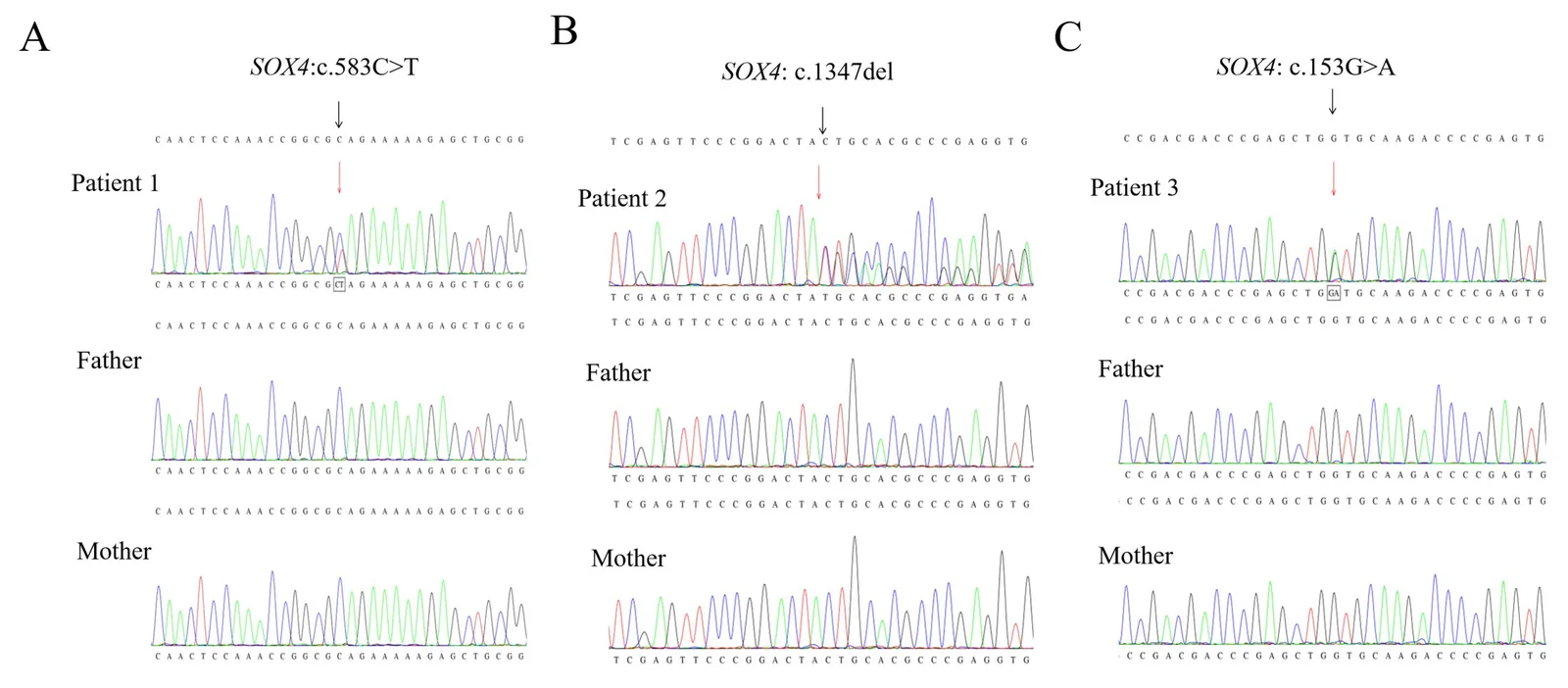
Sanger sequencing chromatograms of probands and parents confirming the de novo origin of *SOX4* variants (NM_003107.3). (**A**) Patient 1: c.583C>T (p.Gln195*); (**B**) Patient 2: c.1347del (p.Cys450Alafs*5); (**C**) Patient 3: c.153G>A (p.Trp51*). Both black and red arrows indicate the variant positions. The chromatograms demonstrate that all variants are absent in all parents. In the Sanger chromatograms, the boxed regions indicate heterozygous peaks, suggesting the concurrent presence of both wild-type and variant alleles at these positions.

Genetic analysis identified three distinct de novo heterozygous truncating variants in *SOX4* (NM_003107.3), including c.583C>T (p.Gln195*), c.1347del (p.Cys450Alafs*5), and c.153G>A (p.Trp51*). No additional candidate variants that could explain the clinical data were identified in the three probands. All three variants were absent from public databases and have not been documented in previous literature. Sanger sequencing subsequently confirmed the selected variants and verified their de novo origin (Figure 1A–C). Pathogenicity was classified according to the ACMG guidelines [13] (Table 1).

### 3.3. Literature Review

**Figure 2:**
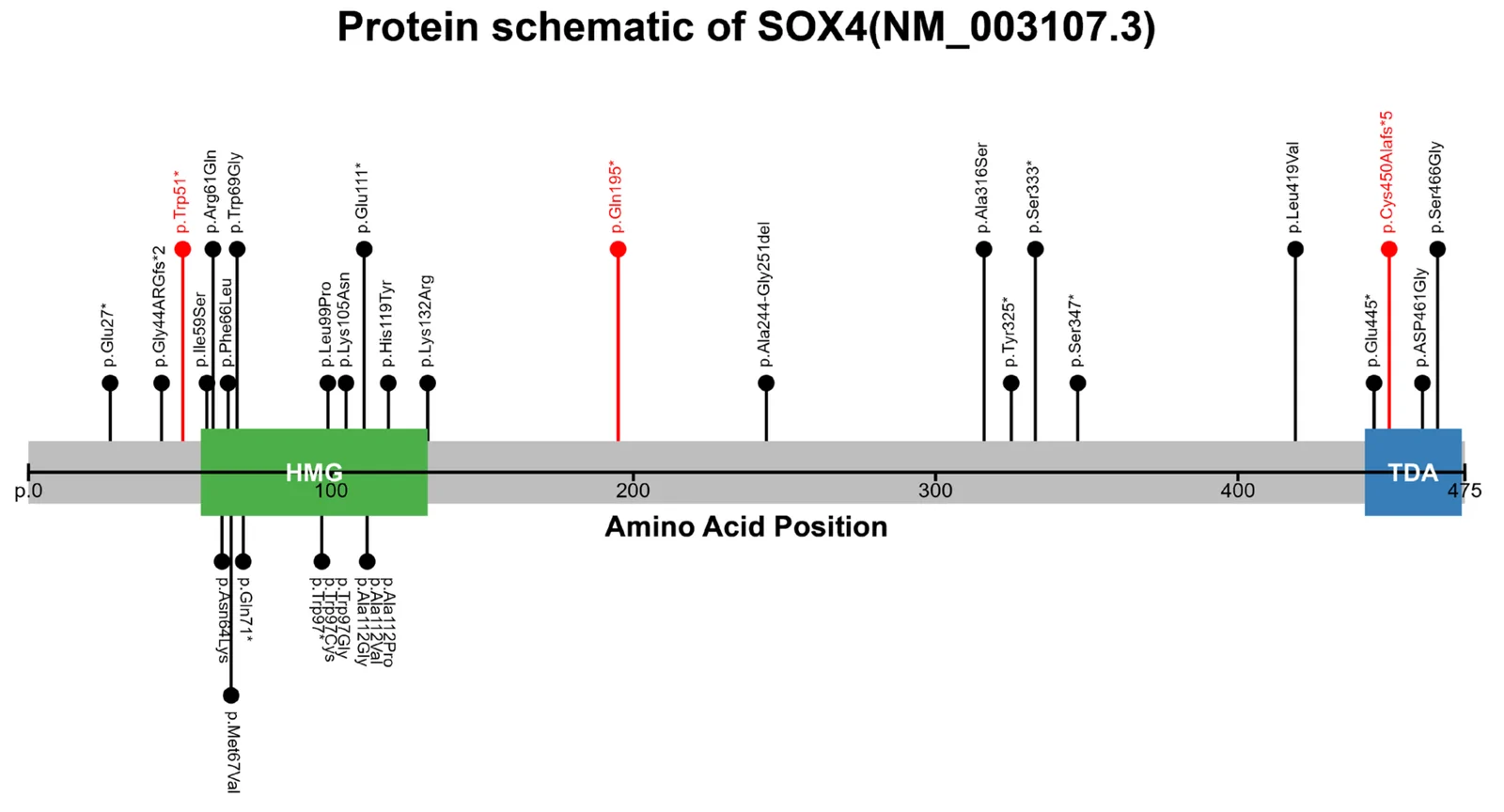
A schematic diagram of SOX4, including the HMG and TAD domains, showing the positions of reported variants at the protein level. Black lollipops represent previously reported cases, while red lollipops represent cases identified in this study. *: stop codon.

As of December 2025, a total of 40 patients affected by *SOX4* have been reported in the literature, involving 29 distinct variants [6,7,8,9,10,11,12]. The variant spectrum includes 19 missense variants (65.5%), 8 nonsense variants (27.6%), 1 frameshift (3.4%), and 1 in-frame deletion (3.4%) (Figure 2). Most variants occur de novo, whereas three recurrent variants showed familial segregation. Specifically, the variant p.Glu111* was identified in a family with five individuals presenting with congenital heart disease, p.Gln71* was detected in a pedigree with seven members affected by atrial fibrillation, and p.Ala244_Gly251del was found in two siblings diagnosed with intellectual disability (Table 2). 

## 4. Discussion

*SOX4*-related disorders, which have been reported in 40 patients to date, are characterized by facial dysmorphism, intellectual disability, developmental delay, cardiac defects, and behavioral abnormalities, with marked clinical heterogeneity [6,7,8,9,10,11,12]. Notably, isolated phenotypes such as atrial fibrillation, congenital heart disease, and dental anomalies have been documented in rare carriers, suggesting that the phenotypic spectrum of *SOX4*-related diseases remains incompletely defined and requires further expansion.

This study reports three patients harboring distinct de novo pathogenic variants in *SOX4*, with clinical phenotypes partially overlapping previously reported features while presenting atypical phenotypic profiles. Consistent across all three patients was speech delay, whereas the remaining clinical manifestations varied substantially. P1 presented with severe hypospadias accompanied by cryptorchidism. P2 had brain dysgenesis, nystagmus, hypotonia, left-sided hearing impairment, and distinctive facial features. P3 manifested global developmental delay with ventricular septal defect. Notably, severe hypospadias with cryptorchidism as the predominant clinical presentation has rarely been reported previously, and nystagmus has not been documented in SOX4-related cases to date. Collectively, these findings further expand the phenotypic spectrum of SOX4-related disorders.

Urogenital anomalies are not typical phenotypes of SOX4-related disorders. To date, only six affected individuals with urogenital involvement have been documented in published cohorts, and cases predominantly presenting with concurrent hypospadias with cryptorchidism are particularly rare [6,7,8,9,10,11,12]. Multiple animal studies have implicated *SOX4* in urogenital development. Murine models have demonstrated that *Sox4* knockout results in elongated gonads in both sexes and increased number of testis cords. Mechanistically, the loss of *SOX4* downregulates the expression of male germ cell differentiation markers (*Nanos2*, *Dnmt3l*) and aberrantly upregulates pluripotency genes (*Cripto*, *Nanog*), suggesting that *SOX4* restricts the duration of pluripotency maintenance in male germ cells, thereby ensuring normal germ cell differentiation [14]. Renal development studies have further confirmed that nephron progenitor-specific deletion of *Sox4* significantly reduces nephron formation, establishing *SOX4* as an indispensable transcription factor for normal kidney development [15]. Furthermore, several SOX family members play pivotal roles in gonadal and external genitalia development. Variants in *SOX9* and its upstream regulatory elements disrupt testis development and induce disorders of sex development [16], whereas structural variants in *SOX3* can cause complete or partial sex reversal in 46,XX individuals [17]. Collectively, these findings support the involvement of *SOX4* in urogenital developmental regulation. Combined with the atypical phenotypic profile of P1, who presented with severe hypospadias and cryptorchidism as the dominant clinical feature, our findings highlight the necessity of careful urogenital evaluation and long-term follow-up in the standardized clinical management of patients with SOX4-related disorders.

To date, ocular anomalies reported in SOX4-related disorders have been limited to myopia and strabismus, and nystagmus has not been previously documented. *SOX4* belongs to the SOXC subfamily, which also includes *SOX11* and *SOX12.* SOXC members serve as essential regulators of ocular structure and visual pathway development. In murine models, retina-specific ablation of either *Sox4* or *Sox11* alone caused only a moderate reduction in retinal ganglion cell (RGC) numbers, whereas their combined deletion resulted in an almost complete loss of RGCs, indicating that the two factors act cooperatively to direct RGC differentiation during early embryogenesis and to maintain RGC survival at later stages [18]. Subsequent studies demonstrated that SOXC proteins bind HES5 and repress Notch signaling, thereby promoting the differentiation of retinal progenitor cells into mature neurons and, in particular, the specification of contralaterally projecting RGCs. Loss of SOXC function results in aberrant ipsilateral projection of RGC axons at the optic chiasm midline [19]. Zebrafish studies have further shown that *sox4* knockdown hyperactivates the Hedgehog pathway, impairs choroid fissure closure, and ultimately induces ocular coloboma [20]. Clinically, sensory-defect infantile nystagmus typically arises from congenital defects of the retina, optic nerve, or other components of the afferent visual pathway. Moreover, nystagmus has also been documented in patients carrying pathogenic variants in other SOX family members, including *SOX2*, *SOX3* and *SOX5*, underscoring the broader involvement of this gene family in ocular development [21,22,23]. Collectively, these findings indicate that *SOX4* loss-of-function variants may trigger nystagmus by disrupting retinal and visual pathway morphogenesis, providing a robust mechanistic basis for the novel nystagmus phenotype identified in our study. Further validation with larger clinical cohorts and functional animal models is required to verify this hypothesis.

*SOX4* maps to chromosome 6p22.3 and encodes a 474-amino-acid transcription factor with two principal functional domains. The high-mobility-group (HMG) box (amino acids 59–132) is responsible for DNA binding, DNA bending, and nuclear translocation, while the C-terminal transactivation domain (TAD, amino acids 442–474) mediates interactions with diverse transcriptional cofactors [7,24,25,26]. To date, a total of 29 *SOX4* variants have been reported, including 19 missense variants (65.5%), 8 nonsense variants (27.6%), 1 frameshift variant (3.4%), and 1 in-frame deletion (3.4%) [6,7,8,9,10,11,12]. Notably, *SOX4* is a single-exon gene; therefore, variants that generate a premature termination codon allow mutant transcripts to escape nonsense-mediated mRNA decay (NMD), leading to the translation of shorter truncated proteins. These truncated proteins may retain partial function or interfere with the normal function of wild-type *SOX4* through a dominant-negative effect [26]. The three patients in this study carried the p.Gln195*, p.Cys450Alafs*5, and p.Trp51* variants, respectively. The p.Gln195* variant results in a 279-amino-acid C-terminal deletion, leading to complete loss of the entire TAD domain. The p.Cys450Alafs*5 variant truncates the C-terminal segment of the TAD, directly impairing transcriptional activation function. In contrast, the p.Trp51* variant is located at the proximal N-terminal region of the open reading frame, generating a truncated protein lacking both the HMG box and TAD. All three truncated proteins lack one or more functional domains, and the distinct sites of truncation are expected to impair protein function to different extents, which may constitute an important molecular basis for the phenotypic heterogeneity observed among our patients.

In this study, we report three unrelated patients harboring three novel de novo truncating variants in *SOX4*, which have not been documented previously. Notably, nystagmus is first incorporated into the phenotypic spectrum of SOX4-associated disorders. Moreover, we describe a unique case characterized by severe hypospadias combined with cryptorchidism as the predominant clinical manifestation. Collectively, this study expands both the mutational and clinical phenotypic spectra of *SOX4*, providing a valuable reference for early recognition of this disorder and genetic counseling for affected families.

## Figures and Tables

**Table 1:** *SOX4* variants and general characteristics of the three patients.

Patient	Gender	Gestational Age	Birth Weight	Age at Presentation	Height	Weight	Position (hg19)	Nucleotide Change (NM_003107.3)	AAchange (NP_003098.1)	GnomAD	SIFT	PolyPhen-2	Mutation Taster	ACMG Classification
P1	M	39^+5^ W	3.5 kg	6 M 1 D	66 cm	7.4 kg	chr6:21595348	c.583C>T	p.Gln195*	0	NA	NA	NA	PVS1 + PS2 + PM2
P2	F	39 W	3.4 kg	1 Y 8 M	81 cm	10 kg	chr6:21596112	c.1347del	p.Cys450Alafs*5	0	NA	NA	NA	PVS1 + PS2 + PM2
P3	F	38 W	2.3 kg	2 M 20 D	55 cm	3.85 kg	chr6:21594918	c.153G>A	p.Trp51*	0	NA	NA	NA	PVS1 + PS2 + PM2

NA = not applicable; *: stop codon; W: week, Y: year, M: month, D: day.

**Table 2:** Clinical features of the present patients and previously reported *SOX4*-related cases.

	This Study	Ghaffar [6]	Angelozzi [7]	Zawerton [8]	Yan [9]	Grosse [10]	Zhou [11]	Jiang [12]	Total Reported Individuals (%)
Total cases	N = 3	N = 2	N = 17	N = 4	N = 5	N = 3	N = 1	N = 8	
Intellectual disability	1/3	2/2	12/12	4/4	5/5	3/3	0/1	1/8	27/35 (77%)
Facial dysmorphisms	1/3	2/2	16/17	4/4	5/5	3/3	1/1	8/8	39/40 (98%)
Speech delay	3/3	1/2	16/17	4/4	0/5	3/3	0/1	0/8	24/40 (60%)
Fifth-finger/toe malformations	0/3	0/2	5/16	4/4	5/5	3/3	1/1	8/8	26/39 (67%)
Cardiac findings	2/3	1/2	8/15	1/4	5/5	2/3	0/1	8/8	25/38 (66%)
Global developmental delay	1/3	2/2	14/16	4/4	0/5	3/3	0/1	0/8	23/39 (59%)
Behavioral concerns	1/3	0/0	15/17	0/0	0/5	3/3	0/1	0/8	18/34 (53%)
Brain anomalies on MRI	1/3	0/2	7/9	2/2	0/5	0/3	0/1	0/8	9/30 (30%)
Hypotonia	1/3	2/2	11/16	2/4	0/5	1/2	0/1	0/8	16/38 (42%)
Ophthalmological findings	1/3	0/2	12/17	1/4	0/5	0/3	0/1	0/8	13/40 (33%)
Ear–nose–throat findings	1/3	0/2	6/16	1/4	0/5	0/3	0/1	0/8	7/39 (18%)
Seizures	0/3	0/2	3/15	1/4	0/5	1/3	0/1	0/8	5/38 (13%)
Genitourinary findings	1/3	0/2	6/16	0/4	0/5	0/3	0/1	0/8	6/39 (15%)
Dental anomalies	0/3	0/2	0/17	0/4	0/5	0/3	1/1	0/8	1/40 (3%)

The denominator indicates the number of cases evaluated for a given feature, while the numerator indicates the number of cases presenting that feature.

## Data Availability

The datasets produced during this investigation are reported in the manuscript. Unprocessed data may be obtained by contacting the corresponding author.
